# Assessing an organizational culture instrument based on the Competing Values Framework: Exploratory and confirmatory factor analyses

**DOI:** 10.1186/1748-5908-2-13

**Published:** 2007-04-25

**Authors:** Christian D Helfrich, Yu-Fang Li, David C Mohr, Mark Meterko, Anne E Sales

**Affiliations:** 1 Northwest HSR&D Center of Excellence, VA Puget Sound Healthcare System, Seattle, Washington, USA; 2 Department of Health Services, University of Washington School of Public Health, Seattle, Washington, USA; 3 Department of Biobehavioral Nursing and Health Systems, University of Washington School of Nursing, Seattle, Washington, USA; 4 Center for Organization, Leadership and Management Research, Department of Veterans Affairs, Boston, Massachusetts, USA; 5 Department of Health Services, Boston University School of Public Health, Boston, Massachusetts, USA

## Abstract

**Background:**

The Competing Values Framework (CVF) has been widely used in health services research to assess organizational culture as a predictor of quality improvement implementation, employee and patient satisfaction, and team functioning, among other outcomes. CVF instruments generally are presented as well-validated with reliable aggregated subscales. However, only one study in the health sector has been conducted for the express purpose of validation, and that study population was limited to hospital managers from a single geographic locale.

**Methods:**

We used exploratory and confirmatory factor analyses to examine the underlying structure of data from a CVF instrument. We analyzed cross-sectional data from a work environment survey conducted in the Veterans Health Administration (VHA). The study population comprised all staff in non-supervisory positions. The survey included 14 items adapted from a popular CVF instrument, which measures organizational culture according to four subscales: hierarchical, entrepreneurial, team, and rational.

**Results:**

Data from 71,776 non-supervisory employees (approximate response rate 51%) from 168 VHA facilities were used in this analysis. Internal consistency of the subscales was moderate to strong (α = 0.68 to 0.85). However, the entrepreneurial, team, and rational subscales had higher correlations across subscales than within, indicating poor divergent properties. Exploratory factor analysis revealed two factors, comprising the ten items from the entrepreneurial, team, and rational subscales loading on the first factor, and two items from the hierarchical subscale loading on the second factor, along with one item from the rational subscale that cross-loaded on both factors. Results from confirmatory factor analysis suggested that the two-subscale solution provides a more parsimonious fit to the data as compared to the original four-subscale model.

**Conclusion:**

This study suggests that there may be problems applying conventional CVF subscales to non-supervisors, and underscores the importance of assessing psychometric properties of instruments in each new context and population to which they are applied. It also further highlights the challenges management scholars face in assessing organizational culture in a reliable and comparable way. More research is needed to determine if the emergent two-subscale solution is a valid or meaningful alternative and whether these findings generalize beyond VHA.

## Background

Organizational culture comprises the fundamental values, assumptions, and beliefs held in common by members of an organization [1]. It is stable, socially constructed, and subconscious. Employees impart the organizational culture to new members, and culture influences in large measure how employees relate to one another and their work environment. Theorists propose that organizational culture is among the most critical barriers to leveraging new knowledge and implementing technical innovation [1].

Health services researchers have frequently used Quinn and Rohrbaugh's [2] Competing Values Framework (CVF) to assess organizational culture and its association with important indicators of healthcare processes and outcomes [3-11]. As a result, scholars have credited (or faulted) organizational culture with contributing to significant differences among healthcare facilities in organizational performance [3], quality improvement implementation [10], patient-care quality and efficiency [12], effectiveness of provider teams [4,5], healthcare provider job satisfaction [4,5], and patient satisfaction [7].

Although instruments based on the CVF are the most frequently used in healthcare research to assess organizational culture [13], there has been limited validation of CVF instruments [1,14]. The only published study conducted in a healthcare setting for the express purpose of CVF model validation was restricted to hospital managers from a single geographic locale [15]. It is not clear whether the same CVF model is viable when applied to non-managers, although they typically constitute the largest portion of an organization's members. Therefore, it is important to understand if an organizational culture instrument is reliable and valid when applied to this group.

The objective of the present study is to test psychometric properties of a CVF instrument administered to a large sample of non-supervisory employees in a large healthcare delivery organization. We chose to focus on employees without supervisory responsibility because this instrument in particular and CVF instruments in general, have not been previously validated among non-managers in health care organizations.

### The Competing Values Framework

**Figure 1:**
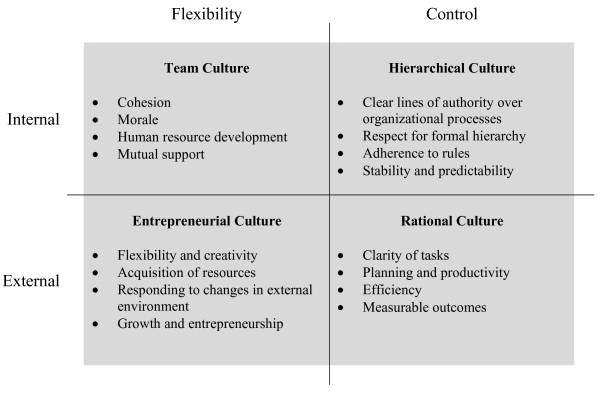
**The competing values framework of organizational effectiveness**. Adapted from: Kalliath, T. J., A. C. Bluedorn and D. F. Gillespie (1999). "A confirmatory factor analysis of the competing values instrument." Educational and Psychological Measurement 59(1): 143–58. Tables.

In the early 1980s, organizational researchers developed the CVF as a conceptual framework to integrate criteria of organizational "effectiveness" [16]. The framework is a synthesis of organizational theories, and posits that most organizations can be characterized along two dimensions, each representing alternative approaches to basic challenges that all organizations must resolve in order to function [17]. The first set of competing values is the degree to which an organization emphasizes centralization and control over organizational processes versus decentralization and flexibility. The second set of competing values is the degree to which the organization is oriented toward its own internal environment and processes versus the external environment and relationships with outside entities, such as regulators, suppliers, competitors, partners and customers. Cross-classifying organizations on these two values dimensions results in four archetypes, referred to as hierarchical, rational, entrepreneurial, and team cultures (Figure 1).

In the CVF, organizations with an internal focus and emphasis on control, labeled hierarchical cultures (also sometimes referred to as "bureaucratic" cultures), adopt centralized authority over organizational processes; respect formal hierarchy; and adhere to rules. They place a premium on stability and predictability. Organizations with an internal focus and emphasis on flexibility, labeled team cultures, encourage broad participation by employees, emphasize teamwork and empowerment, and make human resource development a priority. Organizations with an external focus and emphasis on flexibility, labeled entrepreneurial cultures, exhibit creativity and innovativeness; they place a premium on growth and expanding resources. Finally, organizations with an external focus and an emphasis on control, labeled rational cultures, are characterized by clarity of tasks and goals. They place a premium on efficiency and measurable outcomes.

These four cultures are proposed as archetypes. In reality, organizations are expected to reflect all four cultures to some degree. The CVF does not specify a preferred organizational culture, and there are many competing hypotheses about what cultures or combinations of cultures are superior and under what conditions [18]. However, a fundamental supposition of the CVF is that all four cultures operate at an organizational level and remain relatively stable over time [17]. Furthermore, all four cultures are hypothesized to permeate most facets of the organization, from the comportment of its managers, to the values that bind employees to one another, to the priorities the organization pursues. Therefore, one expects the dominant culture to manifest itself in the views of employees at all levels of the organization [16,17].

The CVF survey instrument most commonly used in health services research comprises 16 items divided equally into four subscales, each representing one of the four archetypal cultures. It was tested initially in three studies of organizational culture in higher education and public utilities [18-20]. The origins of this instrument are unclear: Zammuto and Krakower [20] attribute the instrument to the Institutional Performance Survey developed by the National Center for Education Management Systems in Boulder, CO while Quinn and Spreitzer [19] credit the instrument to Kim S. Cameron. However, most publications cite Zammuto and Krakower [20], who published the complete survey items. A modified 20-item version has been used in health services research [10] and is sometimes referred to as the Quality Improvement Implementation Survey [13,14].

The original 16-item instrument was first validated by Quinn and Spreitzer [19] by means of multi-trait/multi-method analysis and multi-dimensional scaling using survey data from executives of public utilities. The researchers used two versions of the instrument, one with ipsative scales and one with Likert scales. The ipsative or "forced distribution" scales required respondents to allocate 100 points among four survey items according to how well each item described the organization relative to the other items, with each representing one of the four cultures. For example, a respondent might distribute 25 points to team culture, 15 points to entrepreneurial culture, 40 points to bureaucratic culture, and 20 points to rational culture. The Likert scales required respondents to allocate between one and five points per item, independent of how they scored other items. Item wording varied between the two instruments. Quinn and Spreitzer found that data from both versions of the instrument conformed to the CVF, and items among the four subscales correlated, by and large, as predicted in the model. They concluded that the CVF had good construct validity and that the instruments were reliable.

Subsequently, Kalliath and colleagues conducted the only validation of the CVF in a healthcare setting by administering a 16-item, seven-point Likert-scale version of the classic CVF instrument to 300 managers and supervisors from a multi-hospital system in the Midwest [15]. They used structural equations modeling to assess the underlying structure of the survey data to determine if it conformed to the CVF. Their findings were generally consistent with the four-subscale CVF, although they found a high, positive correlation (r = 0.73) between the hierarchical and entrepreneurial subscales, which they anticipated would be uncorrelated or negatively correlated under the CVF. The authors attributed this correlation to the chaotic business environment for hospitals at the time, and concluded that the relationship between the subscales was not fundamentally inconsistent with the CVF.

A significant limitation in both validation studies was their reliance on data exclusively from executives and managers. There are documented gaps in the perceptions of managers versus service providers in areas such as customer expectations [21] and clinic performance [22]; it is conceivable that individuals in supervisory roles may adopt different cognitive maps of organizational values and assumptions than those adopted by rank and file employees. This raises the question to what extent the hypothesized subscales of the CVF emerge among frontline workers in healthcare organizations.

## Methods

In the current study, we analyzed cross-sectional data from a survey of employees of the Veterans Health Administration (VHA) on their work environment, including the organizational culture of their facilities. We conducted three series of analyses on the data. We first conducted item analysis to examine subscale reliability and assess the divergent and convergent properties of the subscales, i.e., the extent to which items correlated within subscales versus across subscales. We then used exploratory factor analysis to determine the underlying structure of the items. Finally, we used confirmatory factor analysis to compare emergent and conventional factor structures. This study was reviewed and approved by the University of Washington Human Subjects Division.

### Data

The 2004 VHA All Employee Survey was distributed to 212,877 VHA employees, including all active clinical, administrative and support staff across all supervisory levels, from frontline workers to senior leaders. Surveys were voluntary and anonymous, and were conducted by a third-party contractor in May 2004. The overall response rate was 51%. Employees had the option of completing surveys online (76% of respondents), by telephone (14%), or by mail (10%).

A total of 75,135 surveys were returned from employees who had no supervisory responsibilities. Because of the large sample size, we elected to exclude 3,359 observations (4.5% of returned surveys) with missing responses for one or more culture items rather than attempting to impute missing data. Compared to respondents who completed all organizational culture items, a higher proportion of respondents with missing items also had missing values on demographic variables such as age, gender and tenure with the VHA (approximately 3% as compared to 1%). Respondents with missing values for organizational culture items also more frequently self-identified as African-American (34% versus 22%) and Hispanic (9% versus 8%), and less frequently as White (53% versus 68%).

The final sample comprised 71,776 surveys returned from employees in 168 VHA facilities who reported having no supervisory responsibility. We used split samples for the exploratory and confirmatory factor analyses [23]. Observations from each of the 168 facilities were randomly assigned to two samples, stratified by facility: a test or model-building sample for the exploratory factor analysis (n = 35,848), and a validation sample for the confirmatory factor analysis (n = 35,928). This ensured roughly equal representation of each facility in both samples, with the facility being the theoretical level of aggregation for the instrument.

### Instrument

The VHA All Employee Survey was fielded by the National Center for Organizational Development in 2004 for organizational development purposes. It included 14 organizational culture items adapted from the Quality Improvement Implementation Survey created by Shortell and colleagues [10]. The Shortell instrument was, itself, an adaptation of the CVF scales reported by Zammuto and Krakower [20]. The latter consisted of 16 items, measuring the four organizational culture archetypes over four organizational domains or dimensions: facility character, cohesion, managers and emphasis. Shortell and colleagues' version [10] added four items (one each for the four cultures) addressing a fifth domain, facility rewards, making 20 items total. The VHA version subsequently dropped the four facility reward items, along with the rational and team culture items from the facility character domain. Items were dropped due to concerns with the length of the overall survey. These six were dropped after pilot testing indicated the items contributed little to scale reliability. Pilot testing also led the VHA survey team to drop the ipsative scales in favor of Likert scales, and to change the wording of two items to improve readability. All three instrument versions are available for comparison, Zammuto and Krakower [20] [see Additional file 1], Shortell and colleagues [10] [see Additional file 2], and the VHA instruments [see Additional file 3].

Respondents scored each item on a five-point Likert scale measuring agreement or disagreement with how well the statement described their facility. For example, the first item states, "My facility is a very dynamic and entrepreneurial place. People are willing to stick their necks out and take risks." A score of one indicates strong disagreement; three indicates neither agreement nor disagreement; and five indicates strong agreement.

### Item analysis

We conducted item analyses to assess the reliability and the convergent/divergent properties of the culture subscales. Subscale reliability and convergent/divergent properties are defined as the extent to which item responses correlate highly within the same subscale and fail to correlate highly with items from across subscales, as predicted by the CVF. We used two sets of measures.

First, we tested the convergent/divergent properties of the items by assessing the item-rest correlation and comparing it to the correlation of the item to each of the three subscales to which it did not belong. Item-rest correlation is the correlation between a given item in a subscale and the aggregate of the remaining items in that subscale. Items should correlate highly with their predicted subscale, demonstrating convergent validity. An item-rest correlation of 0.20 is often considered a minimum acceptable threshold for retaining items in a subscale, and has previously been used for item retention in organizational culture instruments [24]. Items should also correlate lower with other subscales, demonstrating divergent validity. In particular, one would predict that items from orthogonal subscales in the CVF (i.e., the rational versus team subscales, and the hierarchical versus entrepreneurial subscales) should correlate little or not at all. In any case, item-rest correlation should exceed the correlations of the item to the other three subscales to which it does not belong.

Second, we calculated Cronbach's alpha to test internal consistency of items within a subscale [25]. Cronbach's alpha reflects both the length of a scale and the average correlation among items within a scale. A small Cronbach's alpha may suggest that a scale has too few items or the items do not reliably measure a common construct. An alpha of 0.80 or greater is generally considered an indicator of acceptable scale reliability [26].

### Exploratory factor analysis

We conducted exploratory factor analysis to identify emergent factor solutions and determine if the data supported alternative factor solutions. We used principal factor analysis with Promax (oblique) rotation using STATA software (Version 9.2). Principal factor analysis is generally the preferred method for assessing the underlying structures of data [17]. We used oblique rotation, which allows the factors to correlate [27], because the theory underpinning the CVF model anticipates that factors may be correlated [19] and this was consistent with the observed correlations among subscales.

Factors were retained based on three criteria [27]. First, we looked for factors with eigenvalues greater than 1.0. Second, we made a plot of the eigenvalues in descending order to identify the scree, or the point at which the slope of decreasing eigenvalues approaches zero. This indicates the point at which eliminating additional factors would not eliminate significant variance. Third, we retained only factors with two or more items loading at significant levels; we attributed an item to a given factor if the factor loading equaled or exceeded 0.40 [27]. Factors had to meet all three criteria.

### Confirmatory factor analysis

We conducted confirmatory factor analysis to test emergent factor solutions from exploratory factor analysis and compare them with the original four-factor solution to determine which provided a better fit for the data. Confirmatory factor analysis was conducted using weighted least squares (WLS) on polychoric correlation and asymptotic covariance matrices. WLS is usually preferred for analyzing ordinal data because it is more efficient in parameter estimation than other methods, and it corrects standard errors by incorporating weights that are inversely proportional to the variance at each level of the measurement in model fitting [28,29].

We evaluated the model fit using multiple fit indices. The Bentler-Bonnett non-normal fit index (NNFI) [23] and the comparative fit index (CFI) [30] are designed to reflect the goodness of fit of a model independent of sample size. The standardized root mean square residual (SRMR) represents the average absolute value by which observed sample variances and covariances differ from those predicted by the model [31]. Acceptable fit was defined as 0.95 or greater for NNFI and CFI and 0.08 or smaller for SRMR [32]. We also report the Akaike Information Criterion (AIC) which is used to compare models, where smaller values indicate model parsimony [33]. We also report chi-square statistics as an indicator of the overall model fit, with the caveat that the chi-square as a fit index has been criticized for excessive sensitivity in large samples, which may suggest a poor model fit in the absence of true data issues, such as skewness and kurtosis [34]. To provide a metric for the latent constructs, the coefficient of one indicator variable for each of the latent variables was set to 1.0. Based on recommendations by Anderson and West [35], we tested several competing models in which correlations among the latent variables were freely estimated allowing factors to correlate. Analysis was performed using LISREL 8.72 [36].

## Results

Distributions for aggregate scores for all four subscales approximated normal. The overall subscale means ranged from 2.75 (entrepreneurial subscale) to 3.42 (hierarchical subscale). The hierarchical and rational subscales were both left skewed. Subscale scores and indicator scores of individual items were approximately equal for the exploratory and confirmatory samples (Table 1). Although a bivariate test suggested that scores of several items were statistically significantly different between the two samples, this is likely due to the large sample sizes in this study.

**Table 1:** Sample Means for Culture Items and Item Analysis Statistics for Competing Values Framework Subscales.

	EFA sample (n = 35,848)		CFA sample (n = 35,928)			Item correlation to subscales^†^				Cronbach's α
				
	Mean	SD	Mean	SD	p	Entrepreneurial	Hierarchical	Team	Rational	
Entrepreneurial	**2.75**	**0.91**	**2.76**	**0.91**	**0.07**					**0.85**
1. My facility is a very dynamic and entrepreneurial place. People are willing to stick their necks out and take risks.	2.53	1.08	2.54	1.09	0.24	*0.69*	0.17	0.66	0.58	0.80
4. Mangers in my facility are risk-takers. They encourage employees to take risks and be innovative.	2.50	1.08	2.50	1.08	0.70	*0.66*	0.16	0.66	0.59	0.81
8. The glue that holds my facility together is commitment to innovation and development. There is an emphasis on being first.	2.90	1.11	2.91	1.11	0.03	*0.69*	0.27	0.71	0.68	0.80
12. My facility emphasizes growth and acquiring new resources. Readiness to meet new challenges is important.	3.07	1.12	3.08	1.11	0.02	*0.69*	0.28	0.74	0.73	0.80
										
Hierarchical	**3.42**	**0.74**	**3.42**	**0.74**	**0.25**					**0.69**
2. My facility is a very formalized and structured place. Bureaucratic procedures generally govern what people do.	3.51	1.07	3.51	1.07	0.78	0.01	*0.43*	0.05	0.13	0.65
5. Managers in my facility are rule-enforcers. They expect employees to follow established rules, policies, and procedures.	3.67	1.02	3.68	1.01	0.28	0.21	*0.52*	0.26	0.36	0.58
9. The glue that holds my facility together is formal rules and policies. People feel that following the rules is important.	3.45	1.02	3.46	1.01	0.31	0.31	*0.59*	0.36	0.46	0.54
13. My facility emphasizes permanence and stability. Keeping things the same is important.	3.05	1.02	3.05	1.03	0.34	0.23	*0.35*	0.30	0.30	0.70
										
Team	**2.88**	**1.01**	**2.90**	**1.02**	**0.08**					**0.82**
3. Mangers in my facility are warm and caring. They seek to develop employees' full potential and act as their mentors or guides.	2.81	1.20	2.81	1.20	0.63	0.73	0.26	*0.69*	0.69	0.76
7. The glue that holds my facility together is loyalty and tradition. Commitment to this facility runs high.	3.00	1.17	3.02	1.17	0.02	0.68	0.34	*0.65*	0.64	0.79
11. My facility emphasizes human resources. High cohesion and morale in the organization are important.	2.84	1.17	2.86	1.17	0.09	0.74	0.27	*0.71*	0.69	0.73
										
Rational	**3.21**	**0.91**	**3.22**	**0.90**	**0.07**					**0.80**
6. Managers in my facility are coordinators and coaches. They help employees meet the facility's goals and objectives.	3.08	1.14	3.09	1.13	0.59	0.72	0.31	0.77	*0.63*	0.73
10. The glue that holds my facility together is the emphasis on tasks and goal accomplishment. A production orientation is commonly shared.	3.33	1.02	3.34	1.01	0.03	0.58	0.45	0.58	*0.63*	0.73
14. My facility emphasizes competitive actions and achievement. Measurable goals are important.	3.23	1.07	3.24	1.06	0.04	0.66	0.34	0.62	*0.65*	0.71

^† ^These are the Pearson correlations of the individual items to the aggregate of each of the four CVF subscales. For the correlation of the item to its own subscale, we report the item-rest correlation, indicated by italics; the item-rest correlation is simply the correlation of the item to the sum of the rest of the items in that subscale.

Bolded items are statistics for the overall subscale; non-bolded are for the individual item. Cronbach's α for the individual items indicates what the α statistic for that subscale would be were the item in question removed.

To confirm the success of the randomization, we compared the exploratory and confirmatory samples with regard to gender, age, tenure with the VHA, and ethnic and racial background. The samples were virtually identical. Among respondents, 63% were female. Thirty-five percent were between the ages of 50 and 59, and another 33% were between the ages of 40 and 49. Almost 50% of respondents reported being with the VA more than 10 years, and 20% more than 20 years. Sixty-eight percent of respondents self-identified as white, 22% as African American, 5% as Asian, 3% as American Indian/Alaskan and 1% as Hawaiian/Pacific Islander. Across racial groups, 8% percent self identified as Hispanic.

### Item analysis

Item-rest correlations met conventional minimum thresholds of 0.20 for all four subscales, indicating that no individual items had exceptionally poor correlations with their subscales (Table 1). Item correlations to other subscales were also frequently greater than 0.20. In particular, for the entrepreneurial, team, and rational subscales, correlations to other subscales equaled or exceeded the item-rest correlation for eight of the ten items in those subscales, suggesting poor divergent validity.

The entrepreneurial, team, and rational subscales met conventional minimum thresholds for Cronbach's alpha statistics of 0.80, while the hierarchical subscale did not (alpha = 0.69). Next to each item in Table 1, we also report what the Cronbach's alpha for the subscale would be if that item was dropped. For example, dropping item 13 from the hierarchical subscale would minimally improve Cronbach's alpha to 0.70. Dropping any other items from their correspondent subscales would worsen the internal consistency for the subscale.

Overall, item analysis indicated poor convergent/divergent properties for items among the entrepreneurial, team and rational subscales. Among these three subscales, items correlated as high or higher across subscales than within suggesting they collectively may be accounted for by a common underlying factor. Conversely, the hierarchical subscale had a low Cronbach's alpha indicating poor scale reliability. The subscale may include too few items, or items in the subscale may not map onto a single distinct factor. In order to assess the model's overall fit with the data, and to determine if alternative subscales better fit the data, we conducted two sets of factor analyses.

### Exploratory factor analysis

Principal factor analysis revealed a two-factor solution (Table 2). All items from the entrepreneurial, team, and rational subscales loaded significantly on the first factor and items from the hierarchical subscale loaded higher on the second factor. Item ten ("The glue that holds my facility together is the emphasis on tasks and goal accomplishment. A production orientation is commonly shared") loaded higher on the first factor (factor loading of 0.48), although it had a borderline factor loading of 0.39 on the second factor. Items 2 and 13 from the hierarchical subscale had high uniqueness, of 0.73 and 0.80, respectively, indicating 73% and 80% of the observed variance in these two items was not attributable to either of the common factors.

**Table 2:** Factor Loadings from Principal Axis Analysis with Promax Rotation (n = 35,848)

	Factor 1	Factor 2	Uniqueness^†^
Entrepreneurial			
1. My facility is a very dynamic and entrepreneurial place. People are willing to stick their necks out and take risks.	**0.77**	-0.11	0.47
4. Mangers in my facility are risk-takers. They encourage employees to take risks and be innovative.	**0.79**	-0.15	0.46
8. The glue that holds my facility together is commitment to innovation and development. There is an emphasis on being first.	**0.75**	0.06	0.39
12. My facility emphasizes growth and acquiring new resources. Readiness to meet new challenges is important.	**0.78**	0.06	0.34
			
Hierarchical			
2. My facility is a very formalized and structured place. Bureaucratic procedures generally govern what people do.	-0.19	**0.58**	0.73
5. Managers in my facility are rule-enforcers. They expect employees to follow established rules, policies, and procedures.	-0.01	**0.66**	0.57
9. The glue that holds my facility together is formal rules and policies. People feel that following the rules is important.	0.08	**0.72**	0.43
13. My facility emphasizes permanence and stability. Keeping things the same is important.	0.15	0.36	0.80
			
Team			
3. Mangers in my facility are warm and caring. They seek to develop employees' full potential and act as their mentors or guides.	**0.82**	-0.05	0.36
7. The glue that holds my facility together is loyalty and tradition. Commitment to this facility runs high.	**0.68**	0.14	0.44
11. My facility emphasizes human resources. High cohesion and morale in the organization are important.	**0.81**	0.01	0.34
			
Rational			
6. Managers in my facility are coordinators and coaches. They help employees meet the facility's goals and objectives.	**0.77**	0.08	0.35
10. The glue that holds my facility together is the emphasis on tasks and goal accomplishment. A production orientation is commonly shared.	**0.48**	0.39	0.46
14. My facility emphasizes competitive actions and achievement. Measurable goals are important.	**0.61**	0.21	0.46

Note: Factor loadings in bold exceed the conventional threshold of 0.40 for attributing variables to a given factor.

^† ^Uniqueness indicates the proportion of variance unaccounted for by the factors

The items with the highest factor loadings on the first factor were item three ("Managers in my facility are warm and caring. They seek to develop employees' full potential and act as their mentors or guides."), and item 11 ("My facility emphasizes human resources. High cohesion and morale in the organization are important."). Both emphasize supporting employees, fulfilling potential and developing high morale. The items with the lowest factor loadings that still loaded significantly on the first factor were item 14 ("My facility emphasizes competitive actions and achievement. Measurable goals are important."), item seven ("The glue that holds my facility together is loyalty and tradition. Commitment to this facility runs high."), and item eight ("The glue that holds my facility together is commitment to innovation and development. There is an emphasis on being first."). All of the items loading significantly on the first factor emphasize commitment, competitive achievement and fulfilling potential and seem to appeal to a view of organizations as promoting or facilitating human virtues. We label this first factor *humanistic culture*.

Three of four items from the hierarchical subscale loaded onto the second factor. The exception was item 13 ("My facility emphasizes permanence and stability. Keeping things the same is important."), which loaded primarily on the second factor, but had a modest factor loading of 0.36. The three items loading significantly on the second factor emphasize formal rules, bureaucracy and structure. We labeled this second factor *prescriptive culture*.

Because prior validations of the CVF supported a four-factor solution, we conducted an exploratory factor analysis specifying four factors to be extracted from the data. Results from this analysis did not support a four-factor model. The CVF items were primarily loaded on two of the four factors. The factor loadings followed the same pattern presented in Table 2, with slightly lower but salient factor loadings on the correspondent factors. One item each from the other two factors had a loading just under 0.35, while the rest of the items had factor loading less than 0.20.

### Confirmatory factor analysis

Based on findings from the exploratory factor analysis, we tested several factor solutions. We started by testing a two-factor solution and comparing it with the conventional four-factor solution to examine which provided a better fit for the data. The four-factor model comprised all 14 items loading onto the factors proposed in the CVF. The two-factor model comprised 13 items with ten items loading onto humanistic culture and three items on prescriptive culture (dropping Item 13 which failed to load significantly on either factor in the EFA). All models had correlated factors. Results of confirmatory factor analysis are summarized in Table 3.

**Table 3:** Fit Statistics for the Four-Factor and Two-Factor Models (n = 35,928)

	χ^2^	df	χ^2^/df	NNFI	CFI	SRMR	AIC
Four factor model							
14 items	10346	71	145.72	0.93	0.94	0.140	10414.00
							
Two factor model							
13 item, no cross-load	9950	64	155.47	0.93	0.94	0.150	10003.89
13 items, item 10 cross-loaded	8749	63	138.86	0.94	0.95	0.120	8803.67
12 items, no cross-load, item 2 dropped for low reliability	8351	53	157.57	0.94	0.95	0.130	8401.41
12 items, item 10 cross-loaded, item 2 dropped for low reliability	7057	52	135.71	0.95	0.96	0.110	7109.47

Note: NNFI: Non-Normed Fit Index; CFI: Comparative Fit Index; SRMR: Standardized RMR; AIC: Akaike's Information Criterion.

All models used correlated factors.

Results of fitting the conventional four-factor, 14-item model and the two-factor, 13-item model (suggested by the EFA) indicated that neither met the criteria for a satisfactory model fit (Table 3). The NNFI and CFI were slightly under the conventional cutoff of 0.95 and the SRMR were greater than the cutoff of 0.08. Although each item has a substantial loading on its corresponding factor, the majority of standardized residuals were skewed to the negative side, indicating the models overestimated covariance between items. For both models, the reliability estimate (R^2^) of item two was low at approximately 0.25, which is consistent with the exploratory factor analysis results, where its variance not accounted for by the factors was high at 0.73. As expected, the chi-square statistics were highly significant likely due to the large sample size.

Three subscales (entrepreneurial, team, and rational) of the four-factor model were correlated at r = 0.97, suggesting nearly perfect collinearity between these subscales. The hierarchical subscale was moderately correlated with the other subscales, ranging from r = 0.62 to r = 0.73, indicating sufficient independence between this and other subscales. For the two-factor model, the subscales were correlated at r = 0.64. The largest modification index was for the path from the prescriptive culture to item ten. This indicated that we could expect an improvement in model fit by including this path in the model. This is again consistent with findings from exploratory factor analysis where factor loading of item ten was close to 0.40 on the prescriptive culture subscale.

Following these findings, we tested three alternative solutions of the two-factor model: (1) allowing item ten to cross-load on both factors, (2) excluding item two from the model, and (3) allowing item ten to cross-load on both factors and excluding item two from the model.

The solution from the first alternative model (i.e., allowing item ten to cross-load on both subscales) yielded a χ^2 ^reduction of 1,201 at the cost of one degree of freedom, suggesting a significant improvement in model fit. Goodness-of-fit indices were slightly improved from the initial model. The path from the humanistic culture subscale to item ten dropped from 0.86 to 0.62 as that from the prescriptive culture subscale rose from 0.0 to 0.29. The estimated correlation of humanistic and prescriptive culture subscales also dropped slightly from 0.64 to 0.56. Based on the fit indices, the second alternative model (i.e., excluding item two from the model alone) made a relatively smaller difference in the confirmatory factor analysis results.

The third alternative model, allowing item ten to cross-load and dropping item two, achieved a significant improvement in fit. NNFI and CFI derived from this alternative model were both greater than the cutoff score of 0.95, indicating a reasonably good fit between the hypothesized model and the observed data. AIC for this model was the smallest among the models tested, indicating it achieved the most parsimonious representation of the data. At the same time, despite the data being fitted very much ad hoc at this point, the SRMR remained high at 0.11, and numerous large negative residuals were observed in the solution. Both are potentially indicative of model misspecification.

Based on the third model, we examined subscale reliability statistics for humanistic culture and prescriptive culture. Item-rest correlations for humanistic culture items ranged from 0.64 to 0.78, generally exceeding correlations to conventional CVF subscales. The internal consistency of items on the humanistic culture subscale was high, with a Cronbach's alpha of 0.93. Reliability was modest for prescriptive culture, comprising two items from the hierarchical scale and one item from the rational subscale that cross-loaded on both factors, with a Cronbach's alpha of 0.73; item-rest correlations ranged from 0.50 to 0.65. The humanistic and prescriptive cultures had a moderately high, positive and significant correlation (r = 0.60, p < 0.001); in part this is attributable to item 10, which cross-loaded and contributed to both subscales.

## Discussion

We found problems with the convergent/divergent properties of the CVF subscales when applied to a survey of non-supervisory VHA employees. Employees did not appear to distinguish among entrepreneurial, team and rational cultures. Furthermore, the four-item hierarchical subscale had mediocre reliability. These findings could reflect one or more problems with external, internal, and construct validities.

### External validity

The CVF as a model, or the CVF instrument, may not generalize to the VHA, or to non-managers (or to the combination of both). The CVF was validated originally among managers of non-governmental organizations, whereas this study applied it to non-supervisors in VHA, a national, integrated health care delivery system and agency of the federal government.

Preliminary to the analyses reported here, we conducted measurement equivalence/invariance analysis (ME/I) [37] to compare response equivalence among employees at different supervisory levels to determine whether perceptions of organizational culture are systematically differ among organizational members belonging to various organizational hierarchy levels or subgroups. We found essentially identical factor structures to those reported here, but the item response levels differed systematically such that as supervisory level increases, the higher one rates one's organization on items from the entrepreneurial, team and rational subscales and the lower one rates one's organization on items from the hierarchical subscale (details of these analyses are available from the authors). As a result, we elected to focus our present analysis on the instrument's performance among non-supervisors. However, our preliminary ME/I analysis suggests that the instrument performs differently among different supervisory levels within VHA, and further ME/I analysis based on supervisory level is warranted.

We also believe ME/I analyses are needed to assess response equivalence among employees of an organization over time. Time invariance studies are important to determine whether observed differences reflect changes of phenomenon being studied or changes in the relationships between the factors or constructs and their correspondent items [34].

### Internal validity

The instrument used in this study may have contributed to poor internal validity, owing either to measurement problems with the original instruments published by Shortell and colleagues [10] and Zammuto and Krakower [20], or to modifications made to the survey used in VHA. We focus here on describing the modifications to the VHA instrument, and why we believe they do not represent significant threats to internal validity. We then briefly touch on issues related to the original instruments.

There were three modifications to the instrument used in VHA relative to Shortell and colleagues' instrument [10]. First, the VHA instrument had 14 items rather than 20. Six items were dropped during pilot testing due to survey length constraints. The four items addressing facility rewards were dropped (one each from the four culture subscales), and one additional item each was dropped from the team and rational subscales (both relating to the "facility character" domain). The eliminated items were selected to minimize the effect on scale reliability. For example, dropping the two items from the team subscale reduced the alpha coefficient from 0.79 to 0.78, then to 0.76 in the pilot study data. Summary of pilot findings are available upon request from the authors.

It is conceivable though unlikely that shortening the instrument altered its psychometric properties and accounts for our findings. Four of the dropped items, those addressing facility rewards, were not original to the Zammuto and Krakower survey [20], but were added by Shortell and colleagues [10]. Adding the items back would not alter the high correlations among items in the entrepreneurial, rational and team subscales, and it is unlikely they would change the factor structure. Moreover, one would expect dropped items to be reflected in poor alpha statistics. However, the alpha statistics for the team and rational subscales (the subscales from which two items each were dropped) were already reasonably high, and improving them further would not change our conclusions. The hierarchical subscale was the only one of the four with poor reliability, and it is possible that the addition of an item would have improved the alpha coefficient. On the other hand, a hierarchical subscale based on four items is consistent with the Zammuto and Krakower instrument [20] upon which the Shortell instrument is based [10].

The second modification was to the wording of two items, both from the hierarchical subscale scale. The changes were made following pilot testing to improve readability and comprehension. The wording of VHA items was otherwise identical to that of the Shortell instrument [10]. Nonetheless, the changes may have altered the scales' psychometric properties. In the VHA survey, item nine reads, "The glue that holds my facility together is formal rules and policies. People feel that following the rules is important." In the Shortell and colleagues instrument, the first statement is the same, but the second reads, "Maintaining a smooth-running institution is important here." Similarly, item 13 reads, "My facility emphasizes permanence and stability. Keeping things the same is important." Whereas in the Shortell and colleagues instruments, the latter part of item 13 reads, "Efficient, smooth operations are important." Thus, in the modified VHA items, the elements of coordination and operational efficiency are lost and that of rule adherence and stability are reinforced.

These changes may account for the poor reliability of the hierarchical subscale. This is suggested by the fact that dropping item 13 marginally improved reliability. One can speculate that had a fourth item with better item-rest correlation been included, the hierarchical subscale may have met conventional thresholds of reliability.

Nevertheless, these changes fail to account for the poor divergent properties of the rational, entrepreneurial and team subscales. Items for these three subscales were identical in wording to the Shortell version, yet elicited numerous, high cross-scale correlations. In brief, differences in item wording cannot account for the emergence of the humanistic factor.

Although it is beyond the scope of the current paper, it is worth noting that Shortell and colleagues' instrument [10], itself, adapted the wording of items relative to the Zammuto and Krakower instrument [20]. In most cases these changes were minimal. For example, the term "organization" replaced "institution" and "school," and instead of being a "mentor, sage or father/mother figure," managers were "warm and caring, and acted as mentors or guides." However, in some items, the change was more significant and some key terms did not carry over. The clearest example is item six of the rational subscale, which, in Shortell and colleagues' instrument reads, "Managers in my facility are coordinators and coaches. They help employees meet the facility's goals and objectives." In the Zammuto and Krakower survey, the equivalent items reads, "The head of institution D is generally considered to be a producer, a technician, or a hard-driver." Thus, in the revised item, the manager is presented in a more supportive light, while the sense of the manager as a taskmaster is lost. So far as we know, these changes and their effects on instrument reliability and validity have not been the subject of any published work.

The third and final modification to the VHA instrument is the way the scales were scored. Both Shortell and colleague's instrument [10] and the Zammuto and Krakower instrument [20] – as well as most research in health services using the CVF [4,5,8,9,11] – used ipsative scales. These require respondents to allocate 100 points among four statements, each reflecting one of the hypothesized culture types. The VHA instrument used 5-point Likert scales, or normative scales. Ipsative scales, by their nature, are correlated. For example, respondents can only rate one culture stronger by rating weaker one or more of the others. This imposition of interdependence among subscales often inflates reliability statistics [38]. It also makes such data unsuitable for correlation-based statistical modeling, such as factor analysis and regression modeling [19]. Our use of data based on normative scales is therefore not a threat to internal validity, but it may help explain why we find lower reliability for the hierarchical subscale relative to past studies using ipsative scales. We note that although most studies have used ipsative scales, the validation by Quinn and Spreitzer [19] used two versions of the instrument, one with ipsative scales and one normative (Likert) scales, and the subsequent validation by Kaliath [15] also used normative scales. Thus, it is unlikely that our finding of poor divergent validity is primarily due to the normative scales.

There are also potential threats to internal validity originating with the CVF instrument reported by Zammuto and Krakower [20] and carried over in Shortell and colleagues' instrument [10] that we briefly note. First, terms such as "bureaucratic" and "innovative" likely carry normative connotations for lay readers that may overwhelm the technical nuances they are intended to elicit. For example, organizational theorists often use "bureaucracy" in reference to Weber's three principles of the bureaucracy (e.g., fixed and official jurisdiction for roles within the organization) [39], whereas bureaucracy is a popular byword for pathological adherence to rules and the arbitrary exercise of administrative power. Second, most of the original CVF items consist of two declarative statements, often addressing clearly different aspects of culture, such as smooth-running operations and adherence to rules. Respondents may react to each statement differently but are obliged to respond with a single score. This introduces potential measurement error. Third and finally, items were intentionally organized across four organizational domains or content areas: institutional characteristics, institutional leader, institutional "glue" and institutional emphases (and Shortell and colleagues added a fifth: institutional rewards [10]). A major theoretical assumption of the CVF is that organizational culture pervades and unifies the organization across these different domains. Accordingly, there was one item per culture type for each domain. However, it may be that different cultures exist within each of these domains, or that the cultures operate differently in different domains. By using items across different domains to assess a single culture subscale, the instrument may have introduced measurement error.

### Construct validity

There may be poor construct validity for three of the four CVF culture types. Factor analysis indicates that what have been previously described as entrepreneurial, rational and team cultures are accounted for by a single common factor. We find a simplified 12-item, two-factor model fits the data marginally better and more parsimoniously than the classic CVF. More importantly, the convergent/divergent properties of the two-factor solution are superior. This modified two-factor model may provide an alternative to the CVF subscales, and to that end we describe what we believe are the defining characteristics of each factor, which we dub prescriptive culture and humanistic culture.

The prescriptive culture subscale consists of three items, two from the hierarchical subscale (items five and nine) and one from the rational subscale (item ten); the latter cross-loaded on both subscales. In these items, managers are "rule-enforcers;" employees adhere to "formal rules and policies;" and the facility emphasizes "tasks and goal accomplishment." Thus, there is a strong subtext of extrinsic motivation, deriving from the formal policies of the organization and mediated by management. The object of this motivation is to accomplish the employees' tasks in the service of the facility's goals. In the two items from the hierarchical scale that do not load on prescriptive culture, the facility is a "formalized and structured place" where "bureaucratic procedures" govern (item two), and the touchstone is "permanence and stability" and "keeping things the same" (item 13). This suggests that a crucial difference may exist between this emergent factor and the original construct, with the latter including a sense that formal structure is in the service of stability. Nonetheless, three items provide a limited basis for reliably deriving or assessing a construct. Consequently, our outline of the prescriptive culture construct should be viewed as provisional.

Humanistic culture appears to encompass more conceptually diverse qualities, from "warm and caring" to "commitment to innovation and development" to "loyalty and tradition." Nonetheless, the ten items in the subscale share generally positive connotations. They all reflect qualities that one might characterize as human virtues, and which imply that individuals are intrinsically motivated. The organization works to engender loyalty and commitment to innovation, but these values ultimately derive from the individual employees and the survey items suggest impulsion rather than compulsion.

Item ten, originally of the rational subscale, loaded almost equally onto humanistic and prescriptive cultures, with the loading on prescriptive culture falling just short of the threshold of 0.40. Item 10 states, "The glue that holds my facility together is the emphasis on tasks and goal accomplishment. A production orientation is commonly shared." Conceptually, the reference to tasks and goal accomplishment may map more closely to prescriptive culture. The reason for the cross-loading may be lay readers' confusion over the term "production orientation." Had the item referred only to tasks and goal accomplishment, it may have correlated more highly with the prescriptive culture subscale.

The moderately strong, positive correlation between humanistic and prescriptive cultures suggests that VHA employees do not see cultures of intrinsic and extrinsic motivation as mutually exclusive. In fact this supports a central contention among some proponents of the original CVF model, namely that the same organization may simultaneously exhibit qualities of fundamentally competing value systems, and that the "best" organizational culture may be one of equilibrium [17]. We find a timely example of this in a recent study of top-ranked hospitals for acute cardiac care, which simultaneously exhibited a high degree of flexibility (for example, in applying clinical protocols) and a high degree of rigidity (for example, in selecting and pursuing specific performance targets) [40]. Shortell and colleagues also found culture balance related to the number and depth of changes made by teams in chronic care settings [9], and this finding is consistent with Kalliath and colleagues' observed positive correlation between hierarchical and entrepreneurial cultures [15].

Although we chose new labels, the two factors strongly resemble past management theories including Burns and Stalker's "mechanistic" and "organic" organizations [41], and McGregor's "Theory X" and "Theory Y" of management [42]. Mechanistic organizations are said to be characterized by a clear understanding among employees of their performance obligations and what they can expect from the organization in return, clear policies regarding behavior, and an emphasis on chain of command. Organic organizations are characterized by an ethic of diffuse responsibility and decision making such that each employee is expected to do whatever is necessary to get the job done at the time; they rely on shared values and goals to govern behavior rather than specific and extensive rules and instructions. Theory X holds that employees primarily desire stability and security, and require supervision to be productive. Theory Y holds that employees who share the organization's goals will be intrinsically motivated to do their best and will actively seek responsibilities. Humanistic and prescriptive cultures may be iterations of these constructs. In fact, a wry article in the lay press recently proclaimed that virtually all management theory boils down to some version of a dualistic "humanistic" versus "mechanistic" view of organizations [43].

### Directions for future research

Our study raises questions about the validity of a popular instrument based on the CVF when applied to a sample of non-managers. We identify and describe several explanations our findings. We also describe a two-factor scale solution that emerged as an alternative to the conventional four-factor scale. We dub these two factors humanistic and prescriptive. However, our study is not the final word on the CVF, nor is it a sufficient basis to conclude the two-factor solution is a valid or meaningful alternative. Significant additional research is needed.

The first need is for additional analysis of the differences in perception of organizational culture among managers and non-managers. In a measurement equivalence/invariance analysis preliminary to the results reported here, we found that item response varied among supervisory groups. Further analyses of these differences are needed, as well as analysis of how item response varies within organizations over time.

Second, further research is needed on the psychometrics of particular items. We describe potential issues with item wording and structure – derived both from the original CVF instrument and from changes made to the VHA survey – that may account for some of our findings, notably the poor reliability of the hierarchical subscale. There is also need to explore how experiences with different parts of an organization may influence respondents. For example, perhaps employees perceive different cultures in different workgroups or departments: a physician might perceive their internal medicine service as relatively supportive and entrepreneurial, but their human resources department as relatively rule-bound and bureaucratic. If so, it is not clear how respondents answer questions based on the overall organization.

Third and finally, additional research is needed on the emergent two-factor solution both to determine if it is observed in other settings, and whether it is associated with theoretically relevant organizational processes or outcomes, such as performance measures, in order to establish criterion validity.

## Conclusion

The Competing Values Framework has been the most widely used model in health services research to assess organizational culture. It has been offered as an explanation for organizational differences in implementation of quality improvement activities and quality of care. CVF instruments are generally presented as well-validated with reliable, generalizable subscale solutions. They have been frequently fielded among managers under the assumption that the results provide an accurate gauge of culture as experienced by the broader organization. Our findings suggest that one or more of these assumptions are incorrect.

Overall, this study strikes a note of caution in drawing inferences based on aggregated CVF scales when applied to populations where they have not been validated, such as non-managers. Our findings highlight the challenges management scholars and practitioners face in assessing organizational culture in a reliable and comparable way, and underscore the importance of validating organizational culture instruments in each new context they are used.

## Abbreviations

CVF – Competing Values Framework; VHA – Veterans Health Administration; NFI – the Bentler-Bonnett normal fit index; NNFI – the non-normal fit index; CFI – the comparative fit index; SRMR – standardized root mean square residual

## Competing interests

The author(s) declare that they have no competing interests.

## Authors' contributions

CDH conceived of the study and framed the research design, carried out the reliability analyses, assisted with the exploratory factor analysis, interpreted findings, and drafted the manuscript. YFL carried out the exploratory and confirmatory factor analyses, interpreted findings and helped draft the manuscript. DCM helped frame the study, assisted with the confirmatory factor analysis, interpreted findings, and helped draft the manuscript. MM helped frame the study, interpret findings and draft the manuscript. AES helped frame the study, advised on statistical analyses, interpreted findings and helped draft the manuscript. All authors read and approved the final manuscript.

## Supplementary Material

Additional file 1 **Item wording from Original Competing Values Framework instrument**. Source: Zammuto, R. F. and J. Y. Krakower (1991). Quantitative and qualitative studies of organizational culture. Research in organizational change and development. R. W. Woodman and W. A. Pasmore. Greenwich, CT, JAI Press. **5**. Click here for file

Additional file 2 **Item wording from adapted Competing Values Framework instrument used by Shortell and colleagues**. Source: RAND Improving Chronic Illness Care Evaluation: Click here for file

Additional file 3 **Item wording from adapted Competing Values Framework instrument used by the Veterans Health Administration**. Source: 2004 VH All Employee Survey. The complete All Employee Survey is available at: Click here for file
