# Supplementary material for: Assessing an organizational culture instrument based on the Competing Values Framework: Exploratory and confirmatory factor analyses
Source: Implement Sci. 2007 Apr 25;2:13. doi: 10.1186/1748-5908-2-13 (PMC1865551; doi:10.1186/1748-5908-2-13)
Supplement: Additional file 1 — Item wording from Original Competing Values Framework instrument. Source: Zammuto, R. F. and J. Y. Krakower (1991). Quantitative and qualitative studies of organizational culture. Research in organizational change and development. R. W. Woodman and W. A. Pasmore. Greenwich, CT, JAI Press. 5. [file 1748-5908-2-13-S1.doc]

### Item wording from Original Competing Values Framework instrument

Source: Zammuto, R. F. and J. Y. Krakower (1991). Quantitative and qualitative studies of organizational culture. Research in organizational change and development. R. W. Woodman and W. A. Pasmore. Greenwich, CT, JAI Press. **5**.

**1.** **Institutional Characteristics (Please distribute 100 points)**

Institution A is a very personal place. It is like an extended family. People see [*sic*] to share a lot of themselves.

Institution B is very dynamic and entrepreneurial place. People are willing to stick their necks out and take risks

Institution C is very formalized and structured place. Bureaucratic procedures generally govern what people do.

Institution D is very production oriented. A major concern is with getting the job done. People aren’t very personality involved.

**2. Institution Leader (Please distribute 100 points)**

The head of institution A is generally considered to be a mentor, a sage, or a father or mother figure.

The head of institution B is generally considered to be an entrepreneur, an innovator, or a risk taker.

The head of institution C is generally considered to be a coordinator, an organizer, or an administrator.

The head of institution D is generally considered to be a producer, a technician, or a hard driver.

**3. Institution “Glue” (Please distribute 100 points)**

The glue that holds institution A together is loyalty and tradition. Commitment to this school runs high.

The glue that holds institution B together is a commitment to innovation and development. There is an emphasis on being first.

The glue that holds institution C together is formal rules and policies. Maintaining a smooth-running institution is important here.

The glue that holds institution D together is the emphasis on tasks and goal accomplishment. A production orientation is commonly shared.

**4. Institution Emphases (Please distributed 100 points)**

Institution A emphasizes human resources. High cohesion and morale in the school are important.

Institution B emphasizes growth and acquiring new resources. Readiness to meet new challenges is important.

Institution C emphasizes permanence and stability. Efficient, smooth operations are important.

Institution D emphasizes competitive actions, and achievement. Measurable goals are important.
