# Supplementary material for: Assessing an organizational culture instrument based on the Competing Values Framework: Exploratory and confirmatory factor analyses
Source: Implement Sci. 2007 Apr 25;2:13. doi: 10.1186/1748-5908-2-13 (PMC1865551; doi:10.1186/1748-5908-2-13)
Supplement: Additional file 2 — Item wording from adapted Competing Values Framework instrument used by Shortell and colleagues. Source: RAND Improving Chronic Illness Care Evaluation: [file 1748-5908-2-13-S2.doc]

##### SECTION III. CULTURE

**Instructions:**

This set of questions relates to your facility’s culture. The following items contain four descriptions of health care facilities. Please distribute 100 points among the four descriptions depending on how similar each description is to your facility. None of the descriptions is any better than the others; they are just different.

*For example: In question 1, if Facility A seems very similar to mine, B seems somewhat similar, and C and D do not seem similar at all, I might give 70 points to A and the remaining 30 points to B.*

**Facility Character (Please distribute 100 points)**

1. 70

2. 30

3. 0

4. 0

Each should total 100 points.

**1. Facility Character (Please distribute 100 points)**

A. _____ Facility A is a very *personal* place. It is a lot like an extended family. People seem to share a lot of themselves.

B. _____ Facility B is a very *dynamic and entrepreneurial* place. People are willing to stick their necks out and take risks.

C. _____ Facility C is a very *formalized and structured* place. Bureaucratic procedures generally govern what people do.

D. _____ Facility D is very *production oriented*. A major concern is with getting the job done. People aren’t very personally involved.

**Total = 100**

**2. Facility Managers (Please distribute 100 points)**

A. _____ Managers in Facility A are *warm and caring*. They seek to develop employees’ full

potential and act as their mentors or guides.

B. _____ Managers in Facility B are *risk-takers*. They encourage employees to take risks and be innovative.

C. _____ Managers in Facility C are *rule-enforcers*. They expect employees to follow established rules, policies, and procedures.

D. _____ Managers in Facility D are *coordinators and coaches*. They help employees meet the facility’s goals and objectives.

**Total = 100**

**3. Facility Cohesion (Please distribute 100 points)**

A. _____ The glue that holds Facility A together is *loyalty and tradition*. Commitment to this facility runs high.

B. _____ The glue that holds Facility B together is *commitment to innovation and development*. There is an emphasis on being first.

C. _____ The glue that holds Facility C together is *formal rules and policies.* Maintaining a smooth running operation is important here.

D. _____ The glue that holds Facility D together is the emphasis on *tasks and goal accomplishment*.

A production orientation is commonly shared.

**Total = 100**

**4. Facility Emphases (Please distribute 100 points)**

A. _____ Facility A emphasizes *human resources*. High cohesion and morale in the organization are important.

B. _____ Facility B emphasizes *growth and acquiring new resources*. Readiness to meet new challenges is important.

C. _____ Facility C emphasizes *permanence and stability*. Efficient, smooth operations are important.

D. _____ Facility D emphasizes *competitive actions and achievement*. Measurable goals are important.

**Total = 100**

**5. Facility Rewards (Please distribute 100 points)**

A. _____ Facility A distributes its rewards *fairly equally* among its members. It’s important that everyone from top to bottom be treated as equally as possible.

B. _____ Facility B distributes its rewards based on *individual initiative*. Those with innovative ideas and actions are most rewarded.

C. _____ Facility C distributes rewards based on *rank*. The higher you are, the more you get.

D. _____ Facility D distributes rewards based on the *achievement of objectives*. Individuals who provide leadership and contribute to attaining the facility’s goals are rewarded.

**Total = 100**
