# Supplementary material for: Assessing an organizational culture instrument based on the Competing Values Framework: Exploratory and confirmatory factor analyses
Source: Implement Sci. 2007 Apr 25;2:13. doi: 10.1186/1748-5908-2-13 (PMC1865551; doi:10.1186/1748-5908-2-13)
Supplement: Additional file 3 — Item wording from adapted Competing Values Framework instrument used by the Veterans Health Administration. Source: 2004 VH All Employee Survey. The complete All Employee Survey is available at: [file 1748-5908-2-13-S3.doc]

| ***Culture*** |  |  |  |  |  |  |
| --- | --- | --- | --- | --- | --- | --- |
|  |  |  |  |  |  |  |
| **This set of questions relates to your facility’s culture. Please read each statement. Indicate the extent to which you agree or disagree by selecting the appropriate response.** | | | | | | |
|  |  |  |  |  |  |  |
| **Strongly Agree (5)** |  |  |  |  |  |  |
| **Agree (4)** |  |  |  |  |  |  |
| **Neither Agree Nor Disagree (3)** |  |  |  |  |  |  |
| **Disagree (2)** |  |  |  |  |  |  |
| **Strongly Disagree (1)** |  |  |  |  |  |  |
|  |  |  |  |  |  |  |
| **Facility Character** |  |  |  |  |  |  |
|  |  |  |  |  |  |  |
| 1. My facility is a very ***dynamic and entrepreneurial*** place. People are willing to stick their necks out and take risks. | **1** | **2** | **3** | **4** | **5** |  |
|  |  |  |  |  |  |  |
| 2. My facility is a very ***formalized and structured*** place. Bureaucratic procedures generally govern what people do. | **1** | **2** | **3** | **4** | **5** |  |
|  |  |  |  |  |  |  |
| **Facility Managers** |  |  |  |  |  |  |
|  |  |  |  |  |  |  |
| 3. Managers in my facility are ***warm and caring***. They seek to develop employees’ full potential and act as their mentors or guides. | **1** | **2** | **3** | **4** | **5** |  |
|  |  |  |  |  |  |  |
| 4. Managers in my facility are ***risk-takers***. They encourage employees to take risks and be innovative. | **1** | **2** | **3** | **4** | **5** |  |
|  |  |  |  |  |  |  |
| 5. Managers in my facility are ***rule-enforcers***. They expect employees to follow established rules, policies, and procedures. | **1** | **2** | **3** | **4** | **5** |  |
|  |  |  |  |  |  |  |
| 6. Managers in my facility are ***coordinators and coaches***. They help employees meet the facility’s goals and objectives. | **1** | **2** | **3** | **4** | **5** |  |
|  |  |  |  |  |  |  |
| **Facility Cohesion** |  |  |  |  |  |  |
|  |  |  |  |  |  |  |
| 7. The glue that holds my facility together is ***loyalty and tradition***. Commitment to this facility runs high. | **1** | **2** | **3** | **4** | **5** |  |
|  |  |  |  |  |  |  |
| 8. The glue that holds my facility together is ***commitment to innovation and development***. There is an emphasis on being first. | **1** | **2** | **3** | **4** | **5** |  |
|  |  |  |  |  |  |  |
| 9. The glue that holds my facility together is ***formal rules and policies****.* People feel that following the rules is important. | **1** | **2** | **3** | **4** | **5** |  |
|  |  |  |  |  |  |  |
| 10. The glue that holds my facility together is the emphasis on ***tasks and goal accomplishment***. A production orientation is commonly shared. | **1** | **2** | **3** | **4** | **5** |  |
|  |  |  |  |  |  |  |
| ***Continued on next page*** |  |  |  |  |  |  |

| ***Culture continued*** |  |  |  |  |  |  |
| --- | --- | --- | --- | --- | --- | --- |
| **Strongly Agree (5)** |  |  |  |  |  |  |
| **Agree (4)** |  |  |  |  |  |  |
| **Neither Agree Nor Disagree (3)** |  |  |  |  |  |  |
| **Disagree (2)** |  |  |  |  |  |  |
| **Strongly Disagree (1)** |  |  |  |  |  |  |
|  |  |  |  |  |  |  |
| **Facility Emphases** |  |  |  |  |  |  |
|  |  |  |  |  |  |  |
| 11. My facility emphasizes ***human resources***. High cohesion and morale in the organization are important. | **1** | **2** | **3** | **4** | **5** |  |
|  |  |  |  |  |  |  |
| 12. My facility emphasizes ***growth and acquiring new resources***. Readiness to meet new challenges is important. | **1** | **2** | **3** | **4** | **5** |  |
|  |  |  |  |  |  |  |
| 13. My facility emphasizes ***permanence and stability***. Keeping things the same is important. | **1** | **2** | **3** | **4** | **5** |  |
|  |  |  |  |  |  |  |
| 14. My facility emphasizes ***competitive actions and achievement***. Measurable goals are important. | **1** | **2** | **3** | **4** | **5** |  |
